# Supplementary material for: Double burden of malnutrition and its associated factors among adolescents aged (10–19) years in a rural district of Pakistan
Source: PLOS Glob Public Health. 2026 Jun 3;6(6):e0006457. doi: 10.1371/journal.pgph.0006457 (PMC13232956; doi:10.1371/journal.pgph.0006457)
Supplement: Attachment — Submitted filename: Response sheet.docx [file pgph.0006457.s003.docx]

Dear Editor,

I am pleased to submit our manuscript entitled *“Double Burden of Malnutrition and Its Associated Factors among Adolescents Aged 10–19 Years in a Rural District of Pakistan”* for consideration in PLOS Global Public Health.

This study explores the coexistence of undernutrition and overweight/obesity among adolescents in a rural Pakistani district, highlighting sociodemographic and lifestyle factors associated with this double burden. Malnutrition in adolescence has profound implications for growth, educational attainment, and long-term health outcomes, yet evidence from rural South Asia remains scarce. Our findings provide critical insights for designing integrated nutrition interventions and policies tailored to resource-limited rural settings.

We believe this manuscript is well-suited for PLOS Global Public Health because:

- It addresses a pressing global health challenge by documenting the double burden of malnutrition in a rural South Asian context.
- It identifies actionable factors that can inform community-based nutrition programs and adolescent health policies.
- It aligns with the journal’s mission to amplify diverse perspectives and evidence from underrepresented regions.

The manuscript is original, has not been published elsewhere, and is not under consideration by another journal. All authors have approved the final version and consent to its submission.

We respectfully request your consideration of this manuscript for publication. Thank you for your time and attention.

Sincerely,
Dr Jai K Das

Associate Director

Institute for Global Health and Development

Associate Professor

Department of Paediatrics and Child Health

Aga Khan University, Karachi, Pakistan

Email: jai.das@aku.edu

Response sheet

| Reviewer 1 Comments | Responses |
| --- | --- |
| Ensure consistency in reporting numerical results. In the abstract, the percentage for DBM is stated as 22.5%, but in the discussion section, it is written as 22.58%. Please change it so the percentage is consistently 22.5%. | Thank you for highlighting this discrepancy. The percentage has now been standardized to 22.5% throughout the manuscript. |
| Pay attention to the formatting of the letter 'p' denoting p-value. This letter should be italicized. | Thank you for pointing this out. All p‑value notations have been reviewed and corrected to italic formatting. |
| Please check the references(#32). This literature source is not cited in the manuscript text. | Yes, it has been removed from the text. |
| Conduct a thorough proofread of the entire manuscript to avoid grammatical errors and typos, such as the term "over nutrition", which should be written as one word "overnutrition". | Thank you for the suggestion. The manuscript has been thoroughly proofread, and all identified grammatical and typographical issues have been corrected. |
| (Tables): Where is Table 4? In the manuscript, after Table 3, it goes directly to Table 5. Is this a numbering error, and should Table 5 actually be numbered as Table 4 | Thank you for drawing attention to this. The table numbering has been corrected, and the sequence now follows the appropriate order. |
| (Results, line 211): There is an inconsistency in data presentation. In the abstract, the value for underweight is written as “95% CI: 15.0–20.0”. However, in the results section, it is written as “95% CI: 0.15–0.20”. Please correct this notation. | Thank you for identifying this inconsistency. The confidence interval notation has been standardized to match the format used in the abstract. |
| (Results): Strengthen the explanation of how the selection process proceeded from 1,304 households to only 1,185 adolescents being deemed eligible for selection | Thank you for highlighting. Explanation added in the result section. |
| Please add an explanation regarding missing data in this study. Was there any missing data? How much? And how was it handled? | Missing data were minimal (<1% across variables). When the proportion of missing data is very small, global tests for missing completely at random can be unstable and overly sensitive to sparse patterns. Therefore, missingness was assessed using pairwise diagnostics to examine potential associations between missingness and observed variables. These analyses did not suggest systematic patterns of missingness. Accordingly, complete-case analysis was performed.(details added in statistical analysis ) |
| (Discussion): Strengthen the discussion related to Socio-Economic Factors (Working Status). Please check and cite the following article: <https://doi.org/10.52225/narra.v5i1.2049> | We thank the reviewer for this suggestion. The Discussion section has been strengthened by incorporating evidence from a recent case–control study conducted in Batam, Indonesia (doi:10.52225/narra.v5i1.2049), which demonstrates that nutritional outcomes among school-aged children are strongly influenced by socioeconomic, behavioral, and family-level characteristics. This evidence provides contextual support for our finding that adolescent working status is associated with underweight, reinforcing the interpretation that adolescent work may act as a marker of underlying socioeconomic vulnerability. The relevant citation and discussion have been added accordingly. |
| Strengthen the explanation of the limitations faced in this study, as the limitations currently discussed are still insufficient and overly simplistic. | Strengths and limitations have been revised |
| Reviewer 2 Comments | Response |
| The manuscript addresses an important and timely public health issue—the double burden of malnutrition (DBM) among adolescents in rural Pakistan. The introduction is well-structured, provides strong global and regional context, and clearly highlights the knowledge gap in rural Pakistani settings. The justification for focusing on the TMK district is also well explained. | Thank you for your encouraging feedback on the introduction and contextual framing. |
| The methodology is generally sound and clearly presented. The use of a representative multistage cluster sampling approach strengthens the validity of the findings. The sample size is adequate, and the use of standardized WHO anthropometric criteria is appropriate. The statistical analysis using multinomial logistic regression with survey design adjustments is rigorous and suitable for examining the determinants of DBM. Ethical considerations should be clearly stated in the full manuscript, including ethics approval, consent procedures, and data confidentiality. | Thank you for your thoughtful comments. The ethical considerations section has been reviewed and clarified to ensure all required details are included. |
| The results are presented clearly and are supported by the statistical analysis. The prevalence figures for underweight and overweight/obesity are consistent with existing regional trends. The identification of both risk and protective factors is useful for programmatic planning. Confidence intervals are appropriately reported. Some variables approach marginal significance, and this should be acknowledged in the discussion. | Thank you for this observation. A statement acknowledging variables with marginal significance has been added to the discussion. |
| The discussion appropriately interprets the findings in the context of existing literature. The manuscript highlights how lifestyle, gender, hygiene, mental health, and socioeconomic conditions shape adolescent nutritional outcomes. However, the discussion could benefit from deeper reflection on causal pathways and potential confounding factors. Strengths and limitations of the study—such as reliance on cross-sectional data and possible reporting bias—should also be elaborated more fully. | The discussion has been expanded to address the study’s limitations, including its reliance on cross-sectional data and the possibility of reporting bias |
| The conclusions are consistent with the results and emphasize the need for integrated interventions addressing hygiene, mental health, dietary practices, and gender-specific needs. The recommendations are relevant and aligned with public health priorities in low-resource settings. | Thanks |
| This is a well-written and coherent manuscript that meets publication standards in most areas. Minor improvements in clarity, formatting, and elaboration of methodological limitations will strengthen the manuscript further. | Thanks |

**Editors comment**

The double burden of malnutrition does not mean pooling underweight and overweight/obesity into one category. Instead, DBM is demonstrated when both conditions exist in the same population. Therefore, underweight and overweight/obese should be presented as separate categories, and their coexistence in the population indicates the presence of DBM. Avoid pooling the prevalence!

**Response to Editor:**

We thank the editor for the clarification regarding the definition of the double burden of malnutrition (DBM). In response, we have revised the Results section to clarify that DBM is demonstrated by the **coexistence of undernutrition and overnutrition within the same population**, rather than pooling their prevalences. Specifically, the text now reads:

“Among adolescents in the study, 18.3% were underweight (95% CI: 15.0% – 20.0%) and 4.2% were overweight or obese (95% CI: 3.0% – 5.0%). The coexistence of both undernutrition and overnutrition within this population demonstrates the double burden of malnutrition in rural TMK (Table 1).”

We also confirm that **Tables 1, 2, 3 and 4 present underweight, normal weight, and overweight/obese as separate categories**, with no pooling applied. This ensures that the tables and text fully comply with standard definitions of DBM and the editor’s guidance.
