# Supplementary material for: Double burden of malnutrition and its associated factors among adolescents aged (10–19) years in a rural district of Pakistan
Source: PLOS Glob Public Health. 2026 Jun 3;6(6):e0006457. doi: 10.1371/journal.pgph.0006457 (PMC13232956; doi:10.1371/journal.pgph.0006457)
Supplement: Attachment — Submitted filename: response to reviewer.docx [file pgph.0006457.s004.docx]

| comments | response |
| --- | --- |
| Reviewer #1: 1. (Methods): Please clarify which prevalence estimate was used (30.7% or 20.1%) and how they were combined. Was the higher prevalence (30.7%) used to ensure an adequate sample size, or was the 20.1% figure used? | Thank you for this comment. For the sample size calculation, we used the higher prevalence estimate reported in the National Nutrition Survey 2018 (30.7% among boys). Using the higher estimate ensured that the calculated sample size would be sufficiently powered for both sexes. The lower prevalence (20.1% among girls) was not used in the calculation but was reviewed as part of the reported national distribution of underweight |
| 2. (Methods): Please complete the exclusion criteria in the Methods section. Additional criteria such as pregnancy, psychiatric conditions, and chronic diseases were only mentioned in the Results section but were not stated earlier in the Methods. | Thank you for this helpful comment. We agree that the exclusion criteria related to pregnancy, psychiatric conditions, and chronic diseases were not explicitly stated in the Methods section. We have now revised the Methods to clearly outline all inclusion and exclusion criteria. The flow diagram and Results section remain consistent with these updated criteria. |
| (Conclusion): Please further strengthen the conclusion by adding a sentence that explicitly recommends future research, related to the study's limitations that still require deeper exploration. | Thank you for this constructive suggestion. We agree that the conclusion would be strengthened by explicitly highlighting areas for future research, particularly in relation to the study’s limitations. We have now added a sentence recommending future research directions to address gaps that warrant deeper exploration |
| 4. Overall, this manuscript is well-structured and addresses an important public health issue. The authors have been responsive to the previous reviewers' comments, and the revisions have significantly improved the manuscript. The study is methodologically sound, and the findings provide valuable insights into the double burden of malnutrition among adolescents in rural Pakistan. Therefore, I recommend acceptance with minor revisions to strengthen the manuscript further. | Thank you. |
| Reviewer #2: This manuscript presents a methodologically sound and well-executed study on the double burden of malnutrition among adolescents in rural Pakistan. The study design, sampling strategy, anthropometric measurements based on WHO standards, and use of multinomial logistic regression are appropriate and rigorously applied. The statistical analyses support the conclusions drawn. The manuscript is clearly written in standard English, and the discussion appropriately situates the findings within existing literature while acknowledging study limitations. Data availability is adequately addressed in accordance with PLOS policy, and no ethical or publication concerns were identified. Overall, the manuscript meets the publication criteria of PLOS Global Public Health and is suitable for publication. | Thank you. |
