# Supplementary material for: Double burden of malnutrition and its associated factors among adolescents aged (10–19) years in a rural district of Pakistan
Source: PLOS Glob Public Health. 2026 Jun 3;6(6):e0006457. doi: 10.1371/journal.pgph.0006457 (PMC13232956; doi:10.1371/journal.pgph.0006457)
Supplement: Attachment — Submitted filename: Response_sheet_auresp_3.docx [file pgph.0006457.s005.docx]

| Comments | Response |
| --- | --- |
| **Reviewer #1:** 1. (Abstract): Please ensure consistency in the use of terminology. In the results section of the abstract, the term "lack of handwashing" is used, but in the tables/analysis, the variable is referred to as "no soap available/handwash supplies." Please standardize this to avoid confusing the reader. | Thank you for this helpful comment. We agree that consistent terminology is essential for clarity. In response, we have revised the abstract to use the same terminology as in the tables and analysis. Specifically, the term **“lack of handwashing”** has been replaced with **“no soap available/handwash supplies”** throughout the abstract. |
| 2. (Results): Regarding the number of respondents, page 14 (lines 204-205) states that 1,185 households had an eligible adolescent. In the following paragraph (line 209), the analysis was conducted on 1,159 adolescents. What happened to the other 26 adolescents? Please explain this in detail. Figure 1 also only shows 1,185 eligible, and then directly 1,159. There is no "excluded" box with the reasons. | Thank you for highlighting this point. We have now clarified the discrepancy in the Results section and revised Figure 1 to explicitly show the exclusion of these adolescents and the reasons for exclusion. |
| 3. Overall, this article is already very strong after going through several previous revisions. The authors have also addressed the reviewers' comments very well. Perhaps with a few minor improvements in some sections, as I have mentioned above, this article is suitable for publication. | We thank the reviewer for the positive assessment of our manuscript and for acknowledging the improvements made across revisions. |
| **Reviewer #2:** Thank you for the revisions. The authors have adequately addressed the reviewer comments and editorial suggestions. The manuscript has improved substantially and is now suitable for publication. I have no further comments. | We sincerely thank the reviewer for the positive feedback and for recognizing the substantial improvements made to the manuscript. We appreciate the reviewer’s time and valuable input throughout the review process. |
| **Reviewer #3:** The manuscript focuses on a public health matter of importance, the double burden of adolescent undernutrition and overnutrition in rural Pakistan. The author's methodological approach to studying the double burden of malnutrition among the adolescent population and its determinants is comprehensively explained, sound, with a well-designed study and robust statistical analysis. The articulation of the study findings is in accordance with the methods and analysis, and the discussion contributes by bringing perspectives from studies conducted elsewhere.  The authors have fully and satisfactorily addressed the comments from the second review as indicated in the response sheet. | We are grateful to the reviewer for the thoughtful and encouraging evaluation of our study. We appreciate the recognition of the methodological rigor, statistical analysis, and contribution of the discussion, and we thank the reviewer for confirming that all previous comments have been satisfactorily addressed. |
