# Supplementary material for: Double burden of malnutrition and its associated factors among adolescents aged (10–19) years in a rural district of Pakistan
Source: PLOS Glob Public Health. 2026 Jun 3;6(6):e0006457. doi: 10.1371/journal.pgph.0006457 (PMC13232956; doi:10.1371/journal.pgph.0006457)
Supplement: Attachment — Submitted filename: response sheet for editor.docx [file pgph.0006457.s006.docx]

| Comment | Response |
| --- | --- |
| - We note that Figure 1 says "Not competent 25". Should this be 26? Please check and correct as necessary. | Thank you for this observation. The number reported in Figure 1 (n = 25) is correct. The confusion arose due to the label “Not competent,” which was misleading. This category actually refers to households that did not have an adolescent aged 10–19 years and were therefore ineligible, not excluded due to competency.  We have revised the figure to replace “Not competent 25” with “Ineligible households (n = 25)” for clarity and consistency with the study criteria.  To clarify, the 26 exclusions mentioned in the manuscript refer to a separate stage, where adolescents were excluded due to pregnancy (n = 8), psychiatric conditions (n = 3), or chronic diseases (n = 15). These are distinct from the 25 ineligible households at the earlier stage.  We have updated the figure accordingly to avoid any ambiguity. |
|  |  |
